# Screening of antibiotics to obtain axenic cell cultures of a marine microalga *Chrysotila roscoffensis*

**DOI:** 10.3389/fbioe.2023.1218031

**Published:** 2023-05-25

**Authors:** Jiaojiao Liu, Yan Sun, Lin Zhang, Xiaohui Li, Zhichao He, Chengxu Zhou, Jichang Han

**Affiliations:** ^1^ College of Food and Pharmaceutical Sciences, Ningbo University, Ningbo, China; ^2^ Key Laboratory of Marine Biotechnology of Zhejiang Province, School of Marine Sciences, Ningbo University, Ningbo, China; ^3^ Ningbo Institute of Oceanography, Ningbo, China

**Keywords:** *Chrysotila roscoffensis*, antibiotic, axenic, selection marker, sensitivity

## Abstract

Due to high growth rate, outstanding abiotic stress tolerance, and rich value-added substances, *Chrysotila roscoffensis*, belonging to the phylum of Haptophyta, can be considered as a versatile resource for industrial exploitation of bioactive compounds. However, the application potential of *C. roscoffensis* has drawn attention until just recently, and the understanding related to the biological properties of this species is still scarce. For example, the sensitivities of *C. roscoffensis* to antibiotics, which is essential for the verification of heterotrophic capacity and the establishment of efficient genetic manipulation system is still unavailable. Aiming to provide fundamental information for future exploitation, the sensitivities of *C. roscoffensis* to nine types of antibiotics were tested in this study. The results demonstrated that *C. roscoffensis* exhibited relatively high resistances to ampicillin, kanamycin, streptomycin, gentamicin, and geneticin, while was sensitive to bleomycin, hygromycin B, paromomycin, and chloramphenicol. Using the former five types of antibiotics, a bacteria removal strategy was established tentatively. Finally, the axenicity of treated *C. roscoffensis* was confirmed based on a multi-strategy method including solid plate, 16S rDNA amplification, and nuclear acid staining. This report can provide valuable information for the development of optimal selection markers, which are meaningful for more extensive transgenic studies in *C. roscoffensis*. Moreover, our study also paves the way for the establishment of heterotrophic/mixotrophic cultivation modes of *C. roscoffensis*.

## 1 Introduction

*Chrysotila roscoffensis*, previously named as Pleurochrysis carterae, is a unicellular marine microalga belonging to the phylum of Haptophyta (division) (Paudel et al., 2021). As one of the most abundant coccolithophorids in the ocean, *C. roscoffensis* is characterized by its external calcium carbonate (CaCO_3_) shell called coccoliths (Chen et al., 2019). For a long time, *C. roscoffensis* has attracted significant attention due to its huge contribution to global carbon cycles (mainly conferred by its unique calcification process) and important role on marine primary production (Reid et al., 2011; Taylor et al., 2017).

Until just recently, several studies found that *C. roscoffensis* efficiently accumulates various high value-added substances, such as lipids, polyunsaturated fatty acids (PUFAs) (Lee Chang et al., 2016), and fucoxanthin (Ishika et al., 2017; Lourenço-Lopes et al., 2021). For example, in one scaled up cultivation study, *C. roscoffensis* achieved an average dry biomass productivity of 0.19 g·L^−1^d^−1^ with a cellular lipid content of 33% dry cell weight (DCW) when cultivated in outdoor raceway ponds with a semi-continuous cultivation pattern. The authors also found that *C. roscoffensis* was able to withstand extremely high temperature of 41°C and high pH of 11 (Moheimani and Borowitzka, 2006). In another study, C. roscoffensis exhibited a specific growth rate of 0.55 days^−1^ with a mean lipid content of around 34% DCW at 28°C (Rezvani et al., 2019). Moreover, studies also demonstrated that the PUFAs of C. roscoffensis accounted for around 30%–60% of the total fatty acid (FA) (Lee Chang et al., 2016; Kanda et al., 2020). Same with diatoms, haptophytes use fucoxanthin-chlorophyll protein complexes as the light-harvesting systems, indicating that coccolithophorids can accumulate considerable fucoxanthin content (Pajot et al., 2022). Using a semi-continuous cultivation pattern, Okcu et al. (2021) investigated the growth performance and fucoxanthin content of *C. roscoffensis* cultivated under autotrophic conditions (with aeration). The results indicated that *C. roscoffensis* can achieve an average specific growth rate of 0.32 days^−1^ with a biomass productivity of 340.19 mg·L^−1^d^−1^ and fucoxanthin content of 6.88% DCW. Moreover, studies also suggested that the coccoliths of coccolithophores can be considered as an outstanding biomaterial and shows huge applicational potential in various biomedical fields (Moheimani et al., 2012).

Considering the high growth rate, remarkable tolerance to abiotic stress, and potential value-added substances, developing an efficient cultivation strategy is of great interest for improving the growth performance and commercialization potential of *C. roscoffensis*. Microalgae can be produced through three trophic modes: Autotrophic, heterotrophic, and mixotrophic cultivation. Heterotrophic and mixotrophic cultivation can utilize organic compounds as energy sources and are not dependent on light and inorganic carbon, making them more productive than the autotrophic mode (Kandimalla et al., 2016; Hu et al., 2018). Therefore, heterotrophy and mixotrophy have been proposed as potential solutions to overcome the economic constrain of microalgae production at industrial scale (Chen and Walker, 2011; Patel et al., 2019). However, to the best of our knowledge, no investigation on heterotrophic or mixotrophic cultivation of *C. roscoffensis* has been reported to date. One of the main reasons for this situation is the lack of an efficient strategy to obtain axenic culture of *C. roscoffensis*.

Axenic culture is the prerequisite for investigating the mixotrophic/heterotrophic capability of a specific microalga. Nowadays, various methods have been established to eliminate the bacteria contamination from the xenic microalgae cultures, with the antibiotic cocktail being a universal and effective strategy (Cho et al., 2002; Su et al., 2007; Sena et al., 2011; Han et al., 2016; Vu et al., 2018). However, antibiotics are detrimental to both bacteria and microalgae cells (Scholz, 2014). For specific microalgae, and certain types of antibiotics can even cause rapid cell death in specific microalgae at low concentrations. Moreover, the sensitivities of microalgae to given antibiotics vary greatly among different species. As for *C. roscoffensis*, the only available information associated with its resistance to antibiotics was from the study reported by Endo et al., in which the authors employed hygromycin B (Hym B) with a concentration of 2.5 mg·mL^−1^ for positive transformants screening (Endo et al., 2016). All above demonstrate that an antibiotic sensitivity test of *C. roscoffensis* is imperative.

Besides that, antibiotics are the most widely used selection markers for the screening of positive transformants. For example, geneticin (G418), hygromycin, zeocin, and chloramphenicol (Cm) have been frequently adopted to ensure the genetic vehicles deliver exogenous DNA into recipient microalgae cells (Velmurugan and Deka, 2018). Consequently, an antibiotic sensitivity test is also essential for the establishment of genetic manipulation system in *C. roscoffensis*. Very recently, both the nuclear genome of *C. roscoffensis* and the chloroplast genome of *C. dentata* have been completed (Paudel et al., 2021; Meng et al., 2022). Moreover, stable nuclear transformation technology for *C. roscoffensis* has been reported using polyethylene glycol-mediated transfer in 2016 (Endo et al., 2016). Based on these advances, it is likely to develop new and superior *C. roscoffensis* strains with exceptional phenotypes via genetic manipulation in the future (Liu et al., 2015; Zhang et al., 2016; Fayyaz et al., 2020).

In this study, the sensitivities of *C. roscoffensis* to nine types of antibiotics including ampicillin (Amp), bleomycin (Blm), streptomycin (Str), kanamycin (Km), gentamicin (Gm), G418, hygromycin B, paromomycin (Prm), and Cm were evaluated based on cell density, maximal PSII quantum yield (F_v_/F_m_), and biomass. Subsequently, five antibiotics showing relatively low toxicity to microalgae cells were chosen to remove the bacteria from *C. roscoffensis* cultures. Finally, different methods (including bacteria plate, 16S rDNA amplification, and nucleic acids staining) were adopted to assess the bacteria scavenging effect. This report will provide valuable information for the establishment of axenic technique and the construction of genetic engineering strategy in *C. roscoffensis*.

## 2 Material and methods

### 2.1 Microalgae strain and cultivation conditions

The marine microalga of *C. roscoffensis* NMBjih026-8 was obtained from the Microalgae Collection Center of Ningbo University and cultivated using NMB3# medium (Zhang et al., 2022) (prepared by artificial seawater with a salinity of 25°psu) at 23°C ± 1°C with a light intensity of 60°μ mol photons m^−2^ s^−1^ under a light: Dark cycle of 12: 12 h.

### 2.2 Antibiotics and working concentrations

All nine types of antibiotics were purchased from Solarbio (Beijing, China). Stock solution of each antibiotic was set to a concentration of 50 mg·mL^−1^, sterilized via 0.22 µm filter, and maintained at −20°C before using. *C. roscoffensis* at the end of exponential growth phase (optical density at 750 nm, OD_750_ of 0.15) was inoculated into fresh NMB3# medium with an inoculation ratio of 1: 9 (microalgae culture volume: Medium volume) and conducted a batch cultivation. Simultaneously, antibiotics were added into *C. roscoffensis* cultures, and the concentrations were shown in Table 1. Microalgae cultures without antibiotic addition was taken as the control group. Each group was performed with biological triplicates.

**TABLE 1:** Antibiotics and working concentrations.

Antibiotics	Working concentrations (µg mL^−1^)				
	Treatment 1	Treatment 2	Treatment 3	Treatment 4	Treatment 5
Kan	300	600	900	1,200	1,500
Gm	300	600	900	1,200	1,500
Amp	200	400	600	800	1,000
Str	200	400	600	800	1,000
Hym B	100	200	300	400	500
G418	50	100	150	200	250
Cm	50	100	150	200	250
Prm	20	40	60	80	100
Blm	20	40	60	80	100

Notes: Kan, kanamycin; Gm, gentamicin; Amp, ampicillin; Str, streptomycin; Hym B, hygromycin B; G418, geneticin; Cm, chloramphenicol; Prm, paromomycin; Blm, bleomycin.

### 2.3 Sensitivities of *C. roscoffensis* to antibiotics

During the batch cultivation period, OD_750_ of microalgae cultures was measured every 2 days by Thermo Fisher Scientific Microplater Reader (Varioskan LUX, Finland) to represent the cell concentration (Shen et al., 2022; Zhang et al., 2022). At the final cultivation day (Day 10), 10 mL of *C. roscoffensis* cultures collected by Whatman GF/F filters (0.70 µm) were rinsed twice with Milli-Q water and dried to constant weight (60°C for 48 h) to determine the biomass. Moreover, F_v_/F_m_ was monitored every 2 days using WATER-PAM (WALZ, Germany) to investigate the influences of antibiotics on the photoinactivation.

### 2.4 Axenic culture establishment

According to the sensitivities results, five types of antibiotics (Kan, Amp, Str, Gm, and G418 at concentrations of 600, 1,000, 600, 600, and 50 μg·mL^−1^, respectively) showing relatively low toxicity to microalgae cells were employed to scavenge the bacteria from xenic C. roscoffensis cultures. In brief, microalgal colony on solid NMB3# medium (containing 1% agar, without antibiotics addition) was picked into 50 mL liquid medium. After around a week, an antibiotic cocktail composed of five antibiotics stated above was added into the xenic C. roscoffensis cultures. In the following 4 days, the microalgae cultures were mixed thoroughly using a pipettor every day. Subsequently, 5 mL of cultures were transferred into 45 mL of antibiotics-free liquid NMB3# medium to make the microalgae cells recover from antibiotics stress. Then, 100 μL of recovered cultures were plated on solid NMB3# medium containing five antibiotics with concentrations half as that in liquid medium. Around 3 weeks later, visible microalgal colony was picked into liquid NMB3# medium without antibiotics and cultivated for around 2 weeks. Then, the cultures were adopted for further axenicity verification.

### 2.5 Axenic culture assessment

In total, three approaches were adopted to verify the effect of bacteria removal. 1) Bacteria plate, 1 mL of C. roscoffensis cultures were plated onto solid 2216E medium and cultivated at 25°C for 3 days. Then, the presence/absence of bacteria colonies was used to evaluate the axenicity preliminarily. 2) 16S rDNA amplification, 16S rDNA sequences were amplified using a pair of universal primers of 338F (5′–3′, ACT​CCT​ACG​GGA​GGC​AGC​AG) and 1107R (5′–3′, GGGTTGCGCTCGTTGCG) based on the genomic DNA extracted from the microalgae cultures (Xu et al., 2016; Chen et al., 2021). After being sequenced, the data was analyzed via a local BLAST using the chloroplast genome data of *C. dentata* downloaded from NCBI (GenBank accession number MZ819921) (Paudel et al., 2021). 3) Nucleic acids staining, SYBR Green I (Solarbio^®^, China) was added into microalgae cultures with a volume ratio of 1: 100 and incubated for 10 min at room temperature. Then a fluorescence microscope (NiKON, Japan) was adopted to check the presence of potential bacteria (Bruckner and Kroth, 2009; Han et al., 2016).

### 2.6 Statistical analysis

All data was analyzed by one-way analysis of variance (ANOVA) with SPSS 25.0, and exhibited as mean ± SD (*n* = 3). The difference was considered statistically significant when *p*-value was lower than 0.05.

## 3 Result

### 3.1 Impacts of antibiotics on the cell density of *C. roscoffensis*

**FIGURE 1:**
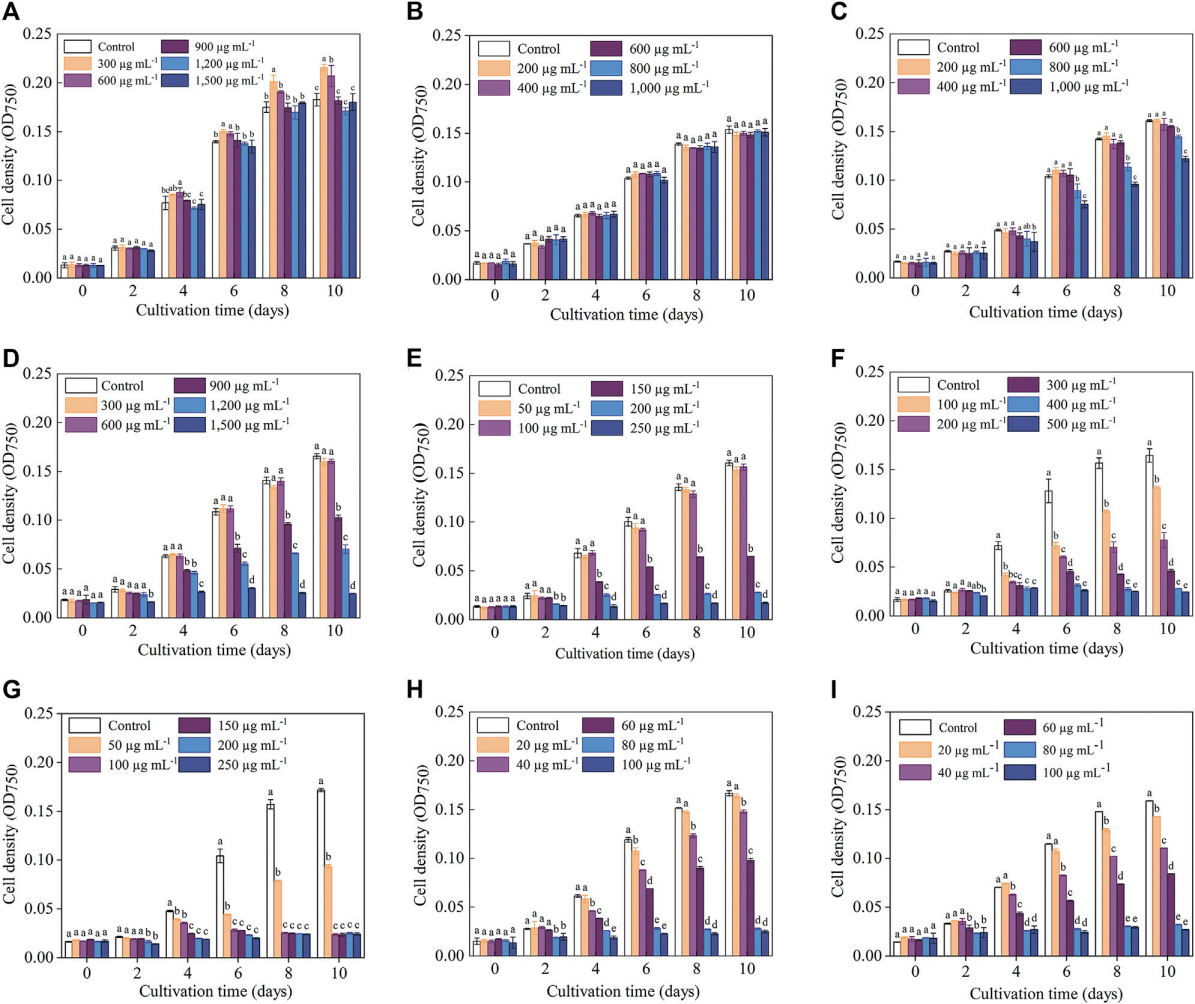
Influence of antibiotics on the growth performance of *C. roscoffensis*. Cell densities of treated by Kan, *kanamycin,* **(A)** Amp, *ampicillin,* **(B)** Str, *streptomycin,* **(C)** Gm, *gentamicin,* **(D)** G418, *geneticin,* **(E)** Hym B, *hygromycin B,* **(F)** Cm, *chloramphenicol,* **(G)** Prm, *paromomycin,* **(H)** and Blm, *bleomycin,* **(I)**.

The cell density data indicated that the sensitivities of *C. roscoffensis* to different antibiotics varied greatly (Figure 1). In general, *C. roscoffensis* showed high resistance to aminoglycosides of Kan, Str, and Gm as well as aminopenicillins of Amp. Especially for Kan, C. roscoffensis at treatments of 900–1,500 μg·mL^−1^ still achieved cell densities similar to the control group. Not only that, Kan at concentrations of 300 and 600 μg·mL^−1^ even exhibited promotion effect on *C. roscoffensis* growth and produced higher final cell densities than the control group (Figure 1A). In terms of Amp, *C. roscoffensis* of all treatments reached similar cell densities to each other during the whole cultivation period (Figure 1B), suggesting a minor effect of Amp on this microalga. As for Str, only the highest two dosages of 800 and 1,000 μg·mL^−1^ resulted in negative influences on the cell density of *C. roscoffensis* (Figure 1C). A similar situation was also obtained from Gm, which only inhibited *C. roscoffensis* growth significantly when the final concentration was higher than 600 μg·mL^−1^ (Figure 1D). G418 displayed a moderate suppression effect on *C. roscoffensis*. With concentrations between 150 and 250 μg·mL^−1^, G418 inhibited microalgae growth sharply, while the treatments of 50 and 100 μg·mL^−1^ showed no significant impacts across the whole cultivation period (Figure 1E).

Compared to above five antibiotics, *C. roscoffensis* displayed relatively low resistances to Cm, Hym B, Prm, and Blm. Hym B at a working concentration of 100 μg·mL^−1^ began to exhibit obvious inhibitory effect on *C. roscoffensis* growth since Day 4, and resulted in a final cell density much lower than the control group (Figure 1F). The strongest inhibition on *C. roscoffensis* growth was observed from Cm, Blm, and Prm (Figures 1H, I). For example, the final cell densities in treatments of Cm at 50 μg·mL^−1^, Blm at 60 μg·mL^−1^, and Prm at 60 μg·mL^−1^ only accounted for around half of the control group. Further increase of concentrations of these three antibiotics even inhibited the growth of *C. roscoffensis* completely.

### 3.2 Effects of antibiotics on the F_v_/F_m_ of *C. roscoffensis*

**FIGURE 2:**
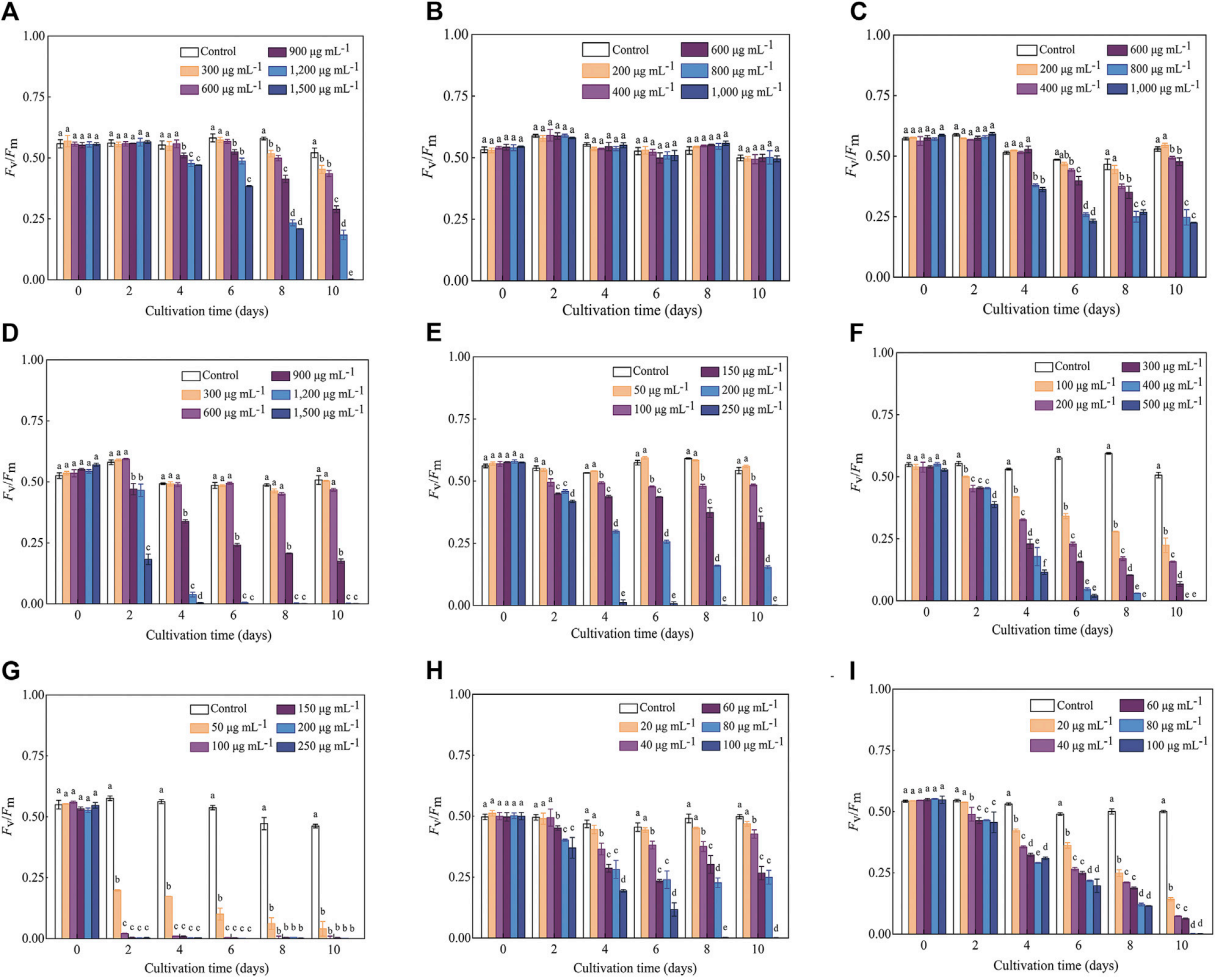
Influence of antibiotics on the *F*_v_/*F*_m_ of *C. roscoffensis*. *F*_v_/*F*_m_ values of treated by Kan, *kanamycin,* **(A)** Amp, *ampicillin,* **(B)** Str, *streptomycin,* **(C)** Gm, *gentamicin,* **(D)** G418, *geneticin,* **(E)** Hym B, *hygromycin B,* **(F)** Cm, *chloramphenicol,* **(G)** Prm, *paromomycin,* **(H)** and Blm, *bleomycin,* **(I)**.

The variation trends of F_v_/F_m_ in response to various antibiotics were generally consistent with those of the cell density, while the inhibitory effect (especially for the treatments with high antibiotics concentrations) reflected in F_v_/F_m_ values occurred earlier than that of the cell density. For example, the OD_750_ values of *C. roscoffensis* under all Kan concentrations remained close to that of the control group throughout the entire cultivation period, while the F_v_/F_m_ values of all Kan treatments were significantly lower than the control group since Day 8. For treatments of Kan with concentrations of 900–1,500 μg·mL^−1^, a significant reduction of F_v_/F_m_ values could even be observed since the fourth day. Not only that, the F_v_/F_m_ value of *C. roscoffensis* treated by 1,500 μg·mL^−1^ nearly declined to 0 at the final cultivation day, suggesting that the microalgae cells were under extreme stress (Figures 1A, 2A). As for Amp, the situation of the F_v_/F_m_ was in line with that of the cell density, confirming that C. roscoffensis possessed outstanding resistance to Amp (Figures 1B, 2B). Regarding Str, treatments of 400 and 600 μg·mL^−1^ showed similar cell densities to the control group during the entire cultivation process, while the F_v_/F_m_ values of these two treatments began to show significant decreases since the sixth day (Figures 1C, 2C). The situations of Gm, G418, and Hym B were the same as that of the Kan and Str. At Day 2, the cell densities of *C. roscoffensis* treated by 900 and 1,200 μg·mL^−1^ of Gm, 100 and 150 μg·mL^−1^ of G418, as well as 100–300 μg·mL^−1^ of Hym B showed no significant differences compared to the control group, while the F_v_/F_m_ values of corresponding treatments started to show obvious inhibitory effects since then (Figures 1D–F; Figure 2D–F). According to the data of cell densities, Cm, Blm, and Prm reached the strongest inhibition to *C. roscoffensis*, and their effects were similar to each other (Figures 1G–I). However, our F_v_/F_m_ data further revealed that the sensitivities of *C. roscoffensis* to these three antibiotics were Cm > Blm > Prm (Figures 2G–I). For example, the F_v_/F_m_ (around 0.2) of *C. roscoffensis* treated by 50 μg·mL^−1^ of Cm sharply decreased to 1/3 of the control group (around 0.58) on Day 2, and the values of other treatments with higher Cm amounts even declined to 0 during the entire cultivation period. According to F_v_/F_m_ data, both Prm and Blm with a concentration of 60 μg·mL^−1^ began to show inhibitory effects on *C. roscoffensis* since Day 2, while their corresponding values were still as high as around 0.45, much higher than that of Cm with a concentration of 50 μg·mL^−1^. Additionally, the F_v_/F_m_ values of Prm with concentrations of 60 and 80 μg·mL^−1^ varied within the range of 0.25–0.35 between the period from Day 4 to Day 10, while that of Blm with the same concentrations gradually dropped below 0.1 on Day 10.

### 3.3 Impacts of antibiotics on the cell density of *C. roscoffensis*

Overall, the inhibitory effects observed on microalgae biomass coincided with those on cell density (Table 2; Figure 1). For example, the biomass of *C. roscoffensis* treated with Kan at concentrations of 1,200 and 1,500 μg·mL^−1^ were 256.7 and 247.2 mg·mL^−1^, respectively, both of which did not differ significantly from the control group (270.0 mg·mL^−1^). All five treatments of Amp achieved final biomass ranging from 223.3 to 233.6 mg·mL^−1^, which were similar to that of *C. roscoffensis* without Amp addition (236.7 mg·mL^−1^). Only the top two, three, and three concentrations of Str, G418, and Gm led to a significant reduction in *C. roscoffensis* biomass. Additionally, Cm, Blm, and Prm also demonstrated the highest inhibitory effects on the biomass accumulation of *C. roscoffensis*, which were consistent with those on cell density.

**TABLE 2:** Effects of antibiotics on biomass of *C. roscoffensis*.

Antibiotics	Biomass (mg L^−1^)					
	Control	Treatment 1	Treatment 2	Treatment 3	Treatment 4	Treatment 5
Kan	270.0 ± 10.2	313.3 ± 14.5*	290 ± 14.5	273.3 ± 20.3	256.7 ± 11.5	247.2 ± 18.6
Gm	233.3 ± 6.7	246.7 ± 6.7	230.0 ± 10.0	160.0 ± 10.7*	106.7 ± 8.8*	50.0 ± 5.8*
Amp	236.7 ± 13.3	233.6 ± 20.3	223.3 ± 3.1	230.0 ± 11.5	231.7 ± 12.0	229.0 ± 15.1
Str	223.2 ± 23.1	223.6 ± 15.3	233.1 ± 27.3	220.2 ± 30.4	180.7 ± 10.9*	171.3 ± 15.5*
Hym B	280.0 ± 10.3	196.7 ± 17.6*	170.0 ± 15.1*	126.7 ± 6.7*	80.0 ± 9.9*	45.0 ± 5.0*
G418	256.7 ± 16.7	246.7 ± 14.5	250.0 ± 11.5	143.3 ± 8.8*	106.7 ± 3.3*	56.7 ± 6.7*
Cm	296.7 ± 14.5	107.0 ± 10.2*	53.7 ± 12.0^*^	50.7 ± 3.3*	47.3 ± 6.1*	50.3 ± 6.7*
Prm	250.0 ± 11.5	243.3 ± 6.7	203.8 ± 8.1*	120.0 ± 10.6*	86.7 ± 10.5*	50.0 ± 5.8*

Notes: Kan, kanamycin; Gm, gentamicin; Amp, ampicillin; Str, streptomycin; Hym B, hygromycin B; G418, geneticin; Cm, chloramphenicol; Prm, paromomycin; Blm, bleomycin. Concentrations of treatments 1–5 are in line with that shown in Table 3. “*” indicates significant difference with the control group.

### 3.4 Axenic culture assessment

According to the results of sensitivity test stated above (mainly based on the F_v_/F_m_ data), Kan, Amp, Str, Gm, and G418 were adopted to scavenge the bacteria from liquid cultures of C. roscoffensis, with working concentrations of 600, 1,000, 600, 600, and 50 µg·mL^−1^, respectively, After recovery from antibiotics, an additional process using the same types but half amount of antibiotics was further conducted to remove the possible residual bacteria from the *C. roscoffensis* colonies on solid NMB3# plates.

**FIGURE 3:**
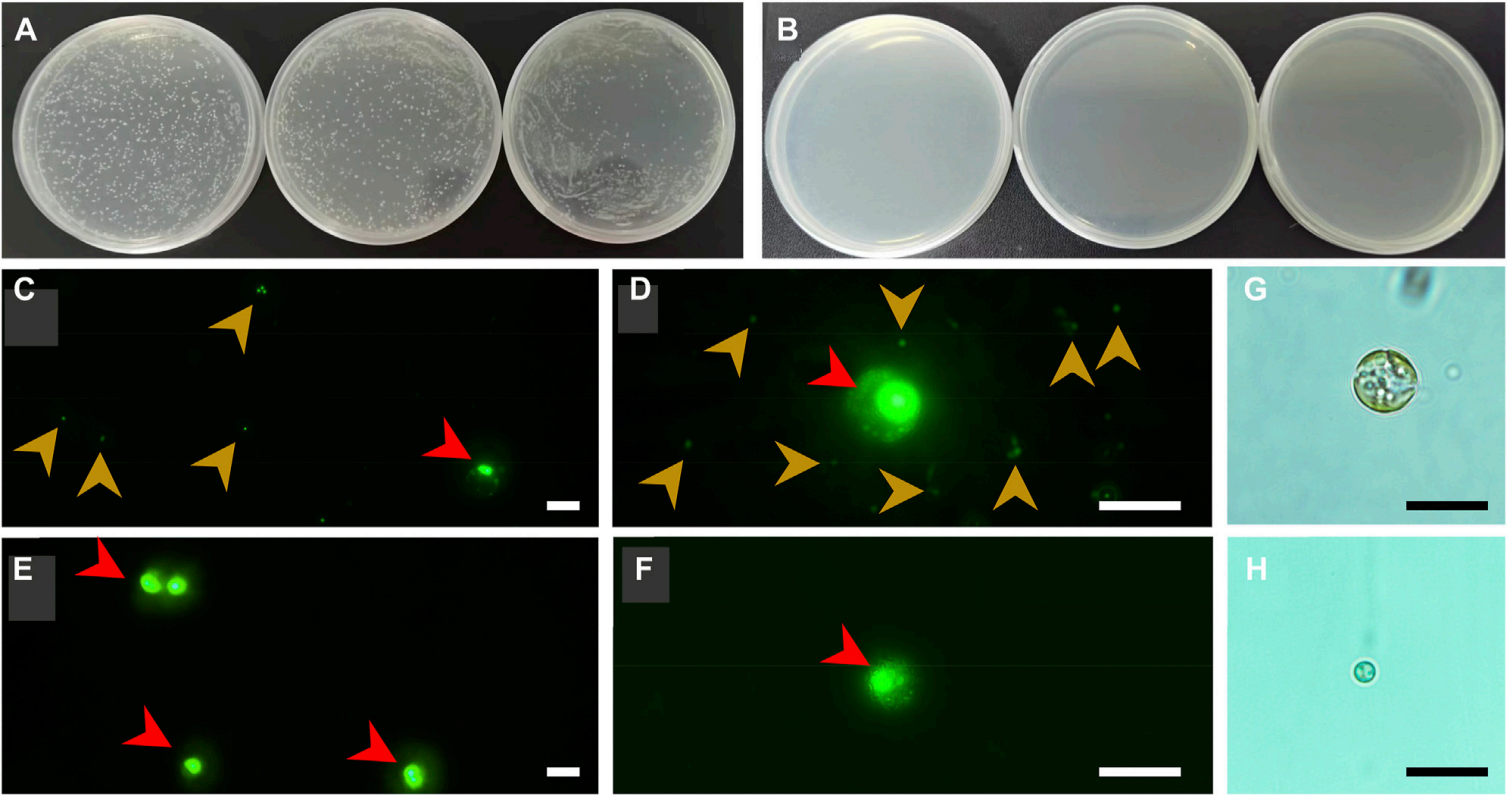
Axenicity verification of *C. roscoffensis*. Solid plates prepared using xenic **(A)** and axenic **(B)**, respectively; **(C, D)** images of xenic cultures under ×40 **(C)** and ×100 **(D)** magnifications, red and yellowish-brown arrows indicate microalgae cells and bacteria, respectively; **(E, F)** images of axenic cultures; **(G, H)** decalcified cells before and after antibiotics treatment. All scale bars in **(C–H)** are10 µm.

Aiming to achieve a reliable conclusion, a multi-strategy method was employed to check the axenicity. Firstly, both axenic and xenic cultures of *C. roscoffensis* were plated on solid agar 2216E medium to evaluate the effectiveness of bacteria removal. As shown in Figures 3A, B, untreated *C. roscoffensis* cultures displayed numerous bacteria colonies, while no visible colony was observed from the plates of treated *C. roscoffensis*.

Using universal primers of 338F and 1107R, a PCR-based strategy was used to identify the presence/absence of potential contaminants. Genomic DNA from axenic *C. roscoffensis* cultures was used as a template, a band around 700 bp was obtained. After sequencing, a BLAST search against NCBI was conducted, and the results indicated that our sequence showed relatively low similarity (with only about 97% identity) with the 16S rDNA sequences of bacteria and cyanobacteria, as well as the plastid 16S rDNA of *Ochrosphaera sp.* and *Braarudosphaera bigelowii* (belonging to *coccolithophorids* same with *Chrysotila spp.*) (Table 3). Considering the chloroplast sequence of *C. dentata* has been published, we further conducted a local BLAST using the plastid genome data of *C. dentata* downloaded from the NCBI and found that our sequence showed a 99.30% identity with the 16S rDNA of *C. dentata*, confirming that our PCR band was amplified from the chloroplast of *C. roscoffensis*. The partial 16S rDNA sequence of *C. roscoffensis* has been submitted to the NCBI with a GenBank No. of OP967475. Besides that, SYBR Green I, a powerful dye for nucleic acids staining, was also adopted to check the presence/absence of bacteria contaminants. As shown in Figures 3C–F, the axenic cultures only showed fluorescence signals with relatively large area (representing the agal cells), while the untreated cultures additionally displayed abundant small green dots suggesting the presence of numerous bacteria in untreated *C. roscoffensis*. All above confirmed that the bacteria contaminants mixed in *C. roscoffensis* have been removed thoroughly by antibiotics.

**TABLE 3:** BLAST results (TOP15 identity) based on the chloroplast 16S rDNA of *C. roscoffensis*.

Organisms	Query cover (%)	E value	Per. Ident (%)	GenBank no.
Marine eukaryote clone 155S3Ab04P	100	0.0	96.78	KX935000.1
Marine bacterioplankton clone E412B_57	100	0.0	96.64	KC003272.1
Bacterium clone N412B_57	100	0.0	96.64	GU940926.1
*Ochrosphaera* sp. 181	100	0.0	96.64	X65101.1
*Braarudosphaera bigelowii*	100	0.0	96.50	AB847986.2
*Braarudosphaera bigelowii*	100	0.0	96.50	AB847985.2
*Braarudosphaera bigelowii*	100	0.0	96.50	AB847984.2
Marine eukaryote clone 155S3Bca4r	100	0.0	96.50	KX937667.1
*Braarudosphaera bigelowii* NIES-4442	100	0.0	96.50	LC595682.1
Cyanobacterium clone F8P4_10C06	100	0.0	96.51	HQ242621.1
Bacterium clone F9P2610_S_H12	100	0.0	96.51	HQ672106.1
Bacterium clone F9P1210_S_H01	100	0.0	96.51	HQ671861.1
Bacterium clone F9P1210_S_D04	100	0.0	96.51	HQ671795.1
Marine bacterium clone A6-3-54	100	0.0	96.51	FJ826362.1
Bacterium clone S25_1594	100	0.0	96.50	EF575250.1

### 3.5 Other general aspects

During the initial period of this study, we suspected that the external coccoliths of *C. roscoffensis* cells may provide a shield for bacteria contaminants, which in turn could have negative effects on subsequent bacteria removal. Therefore, we initially attempted to obtain axenic strains based on protoplasts (Takahashi et al., 2002) rather than intact cells of *C. roscoffensis* at the first beginning time. However, we found that the diameters of *C. roscoffensis* protoplasts decreased gradually from 8–10 µM to 2–3 µM under antibiotics stress (Figures 3G, H). Although the treated protoplasts showed an increased in cell densities when transferred back into antibiotics-free medium, we still gave up this strategy and decided to conduct purification process based on intact cells. Besides that, we also analyzed the growth performances of axenic and xenic *C. roscoffensis* strains, and found that the axenic strain showed a lower growth rate (around 20%) than that of the xenic strain during the first two batches (each batch period lasted for around 2 weeks). However, as the succession progressed, no significant differences associated with the growth rate were observed between axenic and xenic strains.

## 4 Discussion

Axenic cultures are required for verifying the mixotrophy/heterotrophy capacity, genome sequencing (both nuclear and plastid), revealing the microalgal bioactive substances profile, and determining the relationship (beneficial or harmful) between specific bacteria and microalgae for microalgae growth (Borowitzka, 2013; Ramanan et al., 2016; Alvarenga, et al., 2017). Although multiple strategies (e.g., serial dilution, filtration, single-cell isolation, ultraviolet irradiation) have been reported to obtain axenic microalgae strains, an antibiotic cocktail is generally considered as the most commonly used one. Up to now, a great number of antibiotics have been widely adopted to remove bacteria from various microalgae successfully (Table 4) (Han et al., 2016; Cho et al., 2013; Šulčius et al., 2017). All these studies demonstrated that specific microalgae exhibited varied resistances to different antibiotics. Therefore, it is imperative to conduct a sensitive test to determine the proper dosage of antibiotics before they are used for bacteria removal. Besides that, as the most frequently used selection markers in genetic engineering field, antibiotic resistance also plays a crucial role on the screening of positive transformants (Doron et al., 2016; Ng et al., 2017), and thus the investigation of antibiotic sensitivity is also meaningful for the establishment of genetic transformation system in microalgae.

**TABLE 4:** Antibiotics used for axenic purification of microalgae.

Eukaryotic microalgae	Antibiotics and concentrations (μg mL^−1^)	References
*Isochrysis galbana*	Amp 250; Gm 50; Kan 100; Neo 500; Str 50	Cho et al. (2002)
*Alexandrium tamarense*	Gm 100; Str 100; CF 100; Rif 10	Su et al. (2007)
*Karenia mikimotoi*, *Alexandrium tamarense*	Pen 100 U; Str 100; Gm 100; TE 1	Lee et al. (2021)
*Ettlia* sp.	Car 10; Cm 10; Ipm 10; Rif 10; TE 10	Lee et al. (2015)
*Nannochloropsis* sp., *Cylindrotheca* sp., *Tetraselmis* sp., *Amphikrikos* sp.	Amp, Gm, Kan, Neo, Str 6×10^5^	Han et al. (2016)
*Synedra asus*	Cip 5	Shishlyannikov et al. (2011)
*Haematococcus pluvialis*	Gri 100; Amp 5,000	Joo and Lee (2007)
*Amphidinium carterae*	Kan 50; Car 100; Str, 50	Liu et al. (2017)
*Gyrodinium impudicum*	Neo 20; Cep 20	Yim and Lee (2004)
*Nitzschia pungens*	Gm 50; Pen 1,600; Str 800	Douglas and Bates (1992)
*Cymbella microcephala*, *Synedra* acus	Pen 17; Str 8.5; Cm 1.7	Bruckner and Kroth (2009)
*Fragilaria pinnata*, *Synedra ulna*	Pen 170; Str 85; Cm 17; Kan 10; TE 10	Bruckner and Kroth (2009)
*Achnanthes linearis*, *Gomphonema clavatum*	Pen 170; Str 85; Cm 17; TE 0.25; Amp 50	Bruckner and Kroth (2009)
*Navicula cincta*	Pen 170; Str 85; Cm 17; TE 0.25; Amp 50; Kan 2.5	Bruckner and Kroth (2009)
*Cymbella microcephala*	Kan 5; TE 5	Bruckner et al. (2008)
*Haematococcus lacustris*	Aml 2.5	Asatryan et al. (2022)
*Tetraselmis suecica*	VA 5,000; Neo 1×10^4^	Azma et al. (2010)
*Chlorella* sp., *Chlorella sorokiniana*, *Desmodesmus* sp.	Amp 0.5–1×10^5^	Bonett et al. (2020)
*Chlorella* sp., *Monoraphidium* sp.	Ctx 500; TE 50	Tale et al. (2014)

Notes: *Amp, ampicillin;* Kan, *kanamycin; Gm, gentamicin; Neo, neomycin; Str, streptomycin; Cm, chloramphenicol;* Aml, amoxicillin; Car, carbendazim; Ctx, cefotaxime; Cep, cephalosporin; CF, cephalothin; Cip, ciprofloxacin; Gri, griseofulvin; Ipm, imipenem; Pen, penicillin; Rif, rifampicin; Te, tetracycline; VA, vancomycine.

*C. roscoffensis* plays an essential role in global carbon cycles and exhibits potential for value-added substances production. However, its resistances to antibiotics are not well understood, which limits the establishment of heterotrophic/mixotrophic cultivation system and the breeding of superior strain based on genetic manipulation strategy. In this study, the sensitivities of *C. roscoffensis* to nine types of antibiotics, all of which have been widely adopted for both bacteria removal and positive transformants screening in a number of microalgae (Cho et al., 2002; Bruckner and Kroth, 2009; Doron et al., 2016; Velmurugan and Deka, 2018; Lee et al., 2021), were determined according to the cell density, F_v_/F_m_, and biomass weight. The results suggested that *C. roscoffensis* possessed relatively high resistance to Kan, Amp, Str, Gm, and G418, but was sensitive to Cm, Hym B, Prm, and Blm. Therefore, the former five types were chosen to remove the bacteria from *C. roscoffensis* cultures, and the latter four ones were possible to be used as selection markers for genetic transformations in the future.

Cell density, biomass, and F_v_/F_m_ values are widely used as indicators to evaluate the growth performance and metabolic activity of microalgae. Among them, F_v_/F_m_, representing the maximum photochemical quantum yield of photosystem II, is often used to reflect the negative influence of abiotic factors (e.g., nitrogen and phosphate deficiency) on microalgae (Wang et al., 2014; Tan et al., 2019). In this study, our results demonstrated that F_v_/F_m_ can reflect changes in metabolic status in a timely manner. For instance, the F_v_/F_m_ values indicated that Kan at concentrations of 900–1,500 µg·mL^−1^ significantly suppress the metabolism of *C. roscoffensis* since the fourth day. On the contrary, no inhibitory effects caused by Kan were reflected by the cell densities and biomass values during the entire cultivation period. Similar situations also could be found from Str and G418. All these observations suggested that F_v_/F_m_ is more suitable than cell density and biomass for indicating the inhibitory effect of antibiotics on microalgae. To alleviate the negative effects of antibiotics on *C. roscoffensis* as possible, the working concentrations of Kan, Str, and G418 used in this study were determined as 600, 600, and 50 µg·mL^−1^, respectively. Additionally, it should be noted that the relatively low growth rates of axenic strain showed in the first two batches suggest that the antibiotics at these concentrations still result in negative effects to the health of *C. roscoffensis* cells, whereas the potential damage appears to be transient.

A multi-strategy method is always necessary for axenicity confirmation. In general, due to simple operation and low cost, solid plate enriched with organics is a universal method to check the presence of bacteria. However, it has been reported that majority of marine bacteria cannot form visible colonies on solid plate (Guillard, 2005; Bruckner and Kroth, 2009; Vu et al., 2018). Thus, solid agar can only be considered as a strategy for preliminary verification. Alternatively, residual bacteria can be detected using PCR-based methods with universal 16S rDNA primers (Heck et al., 2016). Such a strategy is more sensitive and accurate compared to solid plate, but a sequencing process as well as subsequent peak purity analysis and BLAST search, are always needed, meaning it is relatively time consuming compared to other methods. Moreover, aiming to eliminate false positive results caused by microbial DNA contamination, strict aseptic operation and high-graded reagents are mandatory (Salter et al., 2014). Basically, the method of fluorescence microscopy observation based on SYBR Green I, a safe and membrane-permeable nucleic acid dye possessing strong fluorescence, is recommended as a preferential method. It should be noted that this method may neglect bacteria contaminants when they are in quite low cell density, thus a concentration procedure by centrifuge before staining and observation is necessary.

## 5 Conclusion

In summary, we found that *C. roscoffensis* is relatively resistant to Kan, Amp, Str, Gm, and G418, while Cm, Hym B, Prm, and Blm can significantly inhibit its growth at concentrations below 100 µg·mL^−1^, making them promising selection markers. F_v_/F_m_ is found to be a more suitable indicator of antibiotic sensitivity than cell density or biomass. Using an antibiotic cocktail composed of Kan, Amp, Str, Gm, and G418, we successfully removed bacteria from *C. roscoffensis* cultures, confirmed axenicity using a multi-strategy method, and established an axenic strain for future research. Our next steps will be to investigate the heterotrophy/mixotrophy capacity of *C. roscoffensis* and establish efficient cultivation systems for this valuable microalga.

## Data Availability

The original contributions presented in the study are included in the article/supplementary material, further inquiries can be directed to the corresponding author.
